# Detecting pore-lining regions in transmembrane protein sequences

**DOI:** 10.1186/1471-2105-13-169

**Published:** 2012-07-17

**Authors:** Timothy Nugent, David T Jones

**Affiliations:** 1 Bioinformatics Group, Department of Computer Science, University College London, Gower Street, London, WC1E 6BT, UK

**Keywords:** Transmembrane, Membrane, Protein, Pore, Channel, Prediction, Support vector machine, SVM

## Abstract

**Background:**

Alpha-helical transmembrane channel and transporter proteins play vital roles in a diverse range of essential biological processes and are crucial in facilitating the passage of ions and molecules across the lipid bilayer. However, the experimental difficulties associated with obtaining high quality crystals has led to their significant under-representation in structural databases. Computational methods that can identify structural features from sequence alone are therefore of high importance.

**Results:**

We present a method capable of automatically identifying pore-lining regions in transmembrane proteins from sequence information alone, which can then be used to determine the pore stoichiometry. By labelling pore-lining residues in crystal structures using geometric criteria, we have trained a support vector machine classifier to predict the likelihood of a transmembrane helix being involved in pore formation. Results from testing this approach under stringent cross-validation indicate that prediction accuracy of 72% is possible, while a support vector regression model is able to predict the number of subunits participating in the pore with 62% accuracy.

**Conclusion:**

To our knowledge, this is the first tool capable of identifying pore-lining regions in proteins and we present the results of applying it to a data set of sequences with available crystal structures. Our method provides a way to characterise pores in transmembrane proteins and may even provide a starting point for discovering novel routes of therapeutic intervention in a number of important diseases. This software is freely available as source code from: http://bioinf.cs.ucl.ac.uk/downloads/memsat-svm/.

## Background

Transmembrane channel and transporter proteins are found in the membranes of virtually all organisms and play crucial roles in facilitating the passage of ions and molecules across lipid bilayers. They are essential in maintaining the cross-membrane electrochemical gradient that is essential for a wide variety of fundamental biological processes, from oxidative phosphorylation to signal transduction. For example, voltage-gated ion channels are a class of channel protein that are especially critical for neuron and muscle function. They are activated (i.e., opened) by local changes in electrical potential difference causing an influx of cations, typically sodium or potassium, which induces rapid and coordinated membrane potential depolarisation in response to voltage change, therefore propagating the electrical signal between cells [1,2]. Voltage-gated calcium channels behave similarly but have high permeability to calcium ions when depolarised and are involved in additional pathways including neurotransmitter release at pre-synaptic nerve endings [3]. Aquaporins, members of the major intrinsic protein (MIP) family, are channel proteins that selectively conduct water and small uncharged molecules, such as glycerol, across the membrane [4,5]. They are however completely impermeable to charged species, a property critical for the conservation of the membrane’s electrochemical potential. In mammals, aquaporins are frequently located in the kidney where they play an essential role in water reabsorption. Also found in the kidney is the urea transporter, a specialised channel capable of rapid and selective urea permeation. The crystal structure bears many similarities to ion channels although the presence of a constricted selectivity filter that can accommodate several dehydrated urea molecules in single file confers selectivity for urea [6]. Aside from ions and small molecules, other channels and transporters mediate the transfer of a range of larger biological molecules across the membrane including proteins by the general secretory pathway (SEC) translocase family [7], double-stranded DNA by the septal DNA translocator (S-DNA-T) family [8-10] and mRNA via interactions between the nuclear mRNA exporter (mRNA-E) family and the nuclear pore to facilitate transport into the cytoplasm [11]. Channel and transporter proteins are thus found in a diverse range of cell types and occur as large families of related genes with cell and tissue specific expression patterns. As can be expected, due to their extensive functional diversity, many common diseases including diabetes, hypertension, cardiac arrhythmias, angina pectoris and epilepsy have been related to channel protein dysfunction, therefore transmembrane channels represent one of the most important classes of protein for pharmaceutical intervention [12,13].

Typically, channel proteins contain a cavity (or pore) which spans the entire molecule with an opening on each side of the membrane. The pore often runs parallel to transmembrane helices, forming the path along which ions or molecules travel, with adjacent structural or compositional features determining pore specificity. In many ion channels for example, channel-lining transmembrane helices are enriched with charged residues, thus facilitating passage of the cognate ion through the channel. However, many membrane proteins that are not ion channels contain charged residues within the transmembrane region that are used to stabilise helix-helix interaction, e.g. via formation of salt bridges, thus the presence of charged residues alone cannot be used to discriminate pore-forming regions. Nonetheless, by taking advantage of a number of recent methods that allow the identification of pore-lining residues in membrane protein crystal structures, computational approaches to automatically detect channels are feasible and are likely to provide valuable insight into both structure and function. Pore-Walker [14] identifies the pore centre and pore axis using geometric criteria, allowing the biggest and longest cavity through the channel to be detected. Pore features, including diameter profiles, pore-lining residues, size, shape and regularity can then be calculated.

Here, we present a novel method to automatically predict pore-lining helices in transmembrane proteins. By using Pore-Walker to label pore-lining residues in a training set of transmembrane protein structures, we have employed a supervised learning approach to predict the likelihood of a transmembrane helix being involved in pore formation. Results from testing our method on a crystal structure data set indicate that a prediction accuracy of 72% is possible. Using these predictions as inputs for a support vector regression model, we also show that it is possible to determine pore stoichiometry, i.e. the number of subunits required to form the complete pore, with 62% accuracy.

## Methods

### Data sets

In order to identify pore-lining residues, we used a high resolution data set based on a previously described crystal structure set [15,16], subsequently updated with recent additions to the Orientation of Proteins in Membranes (OPM) database [17] and homology reduced at the 40% sequence identity level. Proteins that lacked either an entry in the Transporter Classification Database (TCDB) [18,19] or Gene Ontology (GO) biological process terms [20] relating to transmembrane transport were then removed. We then used Pore-Walker [14], a fully automated method which detects and characterises channels in transmembrane proteins from their 3D structures, to identify any residues which were exposed to the predicted pore. While other tools such as MOLE [21], CAVER [22], HOLLOW [23] and MolAxis [24] are capable of generating accurate representations of channels in molecular structures, the Pore-Walker algorithm is specifically designed to detect channels and pores in transmembrane proteins by incorporating the typical geometry of membrane proteins, in which the protein’s secondary structures, and therefore the channel, tend to lie perpendicularly to the transmembrane plane. Additionally, these four methods require a priori knowledge of the channel location in order to calculate a route from inside the protein to the outside environment. In a number of cases, Pore-Walker either failed to complete or incorrectly identified the transmembrane plane, resulting in a predicted pore that ran approximately parallel to the membrane plane, while in other cases the pore was completely misaligned with the structure. Where a visual inspection revealed such erroneous pore placement, or where pore placement disagreed with the literature, the protein chain was removed from the data set, resulting in a final training set of 52 chains from 49 protein complexes for which pore-lining residues were identified.

### Machine learning

We used a support vector machine (SVM) classifier to discriminate between the two data classes–residues within the transmembrane region that lined the predicted pore, and residues within the transmembrane region that did not. Input features were generated using evolutionary information. For each sequence in our training set, PSI-BLAST [25] was used to generate position-specific scoring matrices. Two search iterations were performed against the UniRef90 database [26] with a profile-inclusion E-value threshold of 1e-6. We applied a sliding window approach to extract data from the matrix using a window size of 15 centred on the target residue, creating a feature vector of length 300, standardising by Z-score to enable faster SVM convergence (Equation (1)). Only residues from transmembrane helices were used for training, with those predicted as pore-lining labelled as positive examples or negative otherwise. SVM-Light [27] was used for training with a radial basis function kernel, in combination with a grid search of trade-off and gamma parameters, optimising performance using the Matthews Correlation Coefficient (MCC). Stringent cross validation was performed using a jack knife test with the target sequence, along with any other sequences with greater than 25% sequence identity, excluded from training files.

In predicting pore stoichiometry, four features were used to train a support vector regression (SVR) model: sequence length, the number of pore-lining residues, topology and the number of pore-lining helices, with the target value set to the number of subunits contacting the pore within the membrane region. Expressing the fraction of pore-lining residues and pore-lining helices explicitly did not improve performance. Since only a small number of features and training examples were used and therefore training was fast, feature values were not standardised. As with SVM classification, a radial basis function kernel was used with cross-validation carried out using the same protocol. Predicted values were rounded to the nearest integer and the total absolute error across the whole data set was minimised in order to optimise parameters.

## Results and discussion

### Pore-Walker analysis

Analysis of the data set revealed the largest represented TCDB classes were 1.A (alpha-type channels, which catalyse transport via facilitated diffusion), 2.A (porters, which utilise a carrier-mediated process to catalyse uniport, antiport and/or symport) and 3.A (P-P-bond hydrolysis-driven transporters, which drive the active uptake or extrusion of solutes through the hydrolysis of the diphosphate bond of ATP). Members of class 1.A typically had the largest maximum pore diameter although there were exceptions (e.g. where the crystal structure was a ‘closed’ form). In other classes, full pore opening would require some degree of conformational change but in all cases Pore-Walker identified the largest internal cavity from which a narrow path to the opposite side of the membrane could be detected.

The 52 protein chains consisted of 333 transmembrane helices, containing a total of 6688 residues. Of these residues, Pore-Walker identified 1815 within the membrane region as pore-lining, corresponding to 276 transmembrane helices with approximately 40% of residues in a typical helix orientated towards the pore. A significant number of helices contained very few pore-lining residues, therefore in assessing prediction of pore-lining helices, we only included helices containing six or more pore-lining residues, corresponding to approximately a quarter of a typical helix, resulting in a set of 151 pore-lining and 182 non-pore-lining helices. As can be expected for residues with side chains orientated towards a solvent environment, pore-lining helices showed a slight enrichment with polar residues, comprising 19.5% of pore-lining helices, compared to 16.0% of non-pore-lining helices. Most notable was the enrichment of negatively charged aspartic and glutamic acid residues, which constituted 2.1% of residues in pore-lining helices but only 1.2% in helices that did not, and histidine, which can carry a positive charge at physiological pH, accounting for 1.2% of pore-lining residues compared to 0.5%. Pore-lining residue positioning within a helix typically displayed a degree of periodicity, with residues often separated by 3 or 4 positions, corresponding to a complete helical turn indicating that a single face of the helix was orientated towards the pore. This patterning was usually inconsistent over the full length of the helix, suggesting chains did not form a series of ideal helices packed in a tight bundle around the pore, and that helix tilting, twisting or shielding by other helices may play a role.

In order to address the imbalance between data classes, we additionally included residues adjacent to those detected by Pore-Walker in the positive training set; we suggest the inclusion of these residues can be accounted for by dynamics not captured within the crystal structures. This resulted in a class ratio of 3464:3224, which was adjusted to 1:1 by fine-tuning of the SVM cost-factor parameter. Inclusion of these adjacent residues additionally led to a slight increase in per-residue prediction performance (~0.05 MCC). We also attempted including residues in the *i* + 4 and *i−*4 positions to account for those directly above and below the Pore-Walker identified residue in the helix, but this resulted in lower overall performance.

### SVM classifier performance

Table 1 shows the cross-validated performance of the SVM at predicting pore-lining residues and helices. Optimal SVM performance was achieved using a radial basis function gamma value of 0.1 and a trade-off value of 1. The ratio between the number of support vectors in the final model and the number of training examples was approximately 5:7, somewhat higher than the equivalent value when discriminating between e.g. transmembrane helix and loop regions (2:5), reflecting both the relative difficulty of the predictive task and the small size of the training set. To assess prediction of pore-lining helices, the average raw SVM score was determined for residues forming the helix, and a slightly elevated threshold (0.3) was used to optimally identify such helices. At thresholds between 0 and 0.3, it appears that incorrect pore-lining residue predictions are contributing excessively to the prediction of false positive pore-lining helices, therefore this raised threshold in effect helps to negate errors in the prior SVM prediction step by reducing the false positive rate from 0.39 (threshold = 0) to 0.16 (threshold = 0.3). We defined transmembrane helix boundaries using MEMSAT-SVM [15] predictions performed under full cross validation, with the target sequence, along with any other sequences with greater than 25% sequence identity, excluded from training files. We required an overlap of 10 residues between predicted and known helices for correct predictions. Predicted helices that did not overlap with known helices where recorded as false positives if predicted as pore-lining, but were otherwise not recorded as true negatives. In order to provide users with a measure of likelihood in the range 0–1, average raw SVM scores were then converted to Z-scores by subtracting the mean and dividing by the standard deviation of scores from the test set (Equation (1)). Z-scores were used to calculate a posterior probability for each helix by fitting against a standard logistic function. For comparison, we also developed two naïve classifiers that regarded all polar residues as pore-lining, and helices as pore-lining if they contained 7 or more polar residues. These naïve methods were also assessed using transmembrane helix boundaries predicted by MEMSAT-SVM.

(1) $$z=\frac{x-\mu }{\sigma }$$

**Table 1:** SVM performance in predicting pore-lining residues, pore-lining helices and discriminating between monomeric and multimeric pore stoichiometry

**Classifier**	**Precision**	**Recall**	**TPR**	**FPR**	**MCC**	**Accuracy**
Pore-lining residue SVM	0.64	0.54	0.69	0.39	0.30	65%
Pore-lining residue (naïve)	0.53	0.23	0.23	0.21	0.03	51%
Pore-lining helix SVM	0.74	0.35	0.57	0.16	0.43	72%
Pore-lining helix (naïve)	0.50	0.17	0.22	0.17	0.15	55%
Monomeric/multimeric pore stoichiometry SVR	0.88	0.49	0.92	0.11	0.80	90%

TPR: True positive rate. FPR: False positive rate. Pore-lining helices were identified by averaging the raw SVM scores for each participating residue, while a threshold of 7 or more polar residues was found to be optimal for the naïve method.

Equation 1. Z-score calculation*. x* is the raw score, μ is the mean and σ is the standard deviation.

### Identifying pore-lining residues

**Figure 1:**
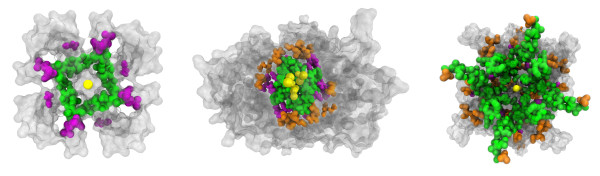
**Prediction of pore-lining residues from sequence mapped onto native crystal structures.** Homotetrameric calcium-gated potassium channel MthK (left, 1LNQ) bottom-up view; no false positives were predicted. Homodimeric molybdate transporter ModB2C2 (centre, 2ONK) top-down view. Homotetrameric AMPA-subtype glutamate receptor (3KG2, right) bottom-up view. The pore identified by Pore-Walker is shown by yellow spheres, while residues correctly predicted as pore-lining are shown in green (true positives), incorrectly predicted as pore-lining are shown in orange (false positives), correctly predicted as non-pore-lining are shown in grey (true negatives) and incorrectly predicted as non-pore-lining are shown in magenta (false negatives).

Results indicate that, while the prediction of pore-lining residues from sequence is generally challenging with a maximum MCC of 0.30, our SVM-based method improves significantly over naïve approaches by up to an order of magnitude. Chains where prediction accuracy was highest included many ion channels, ammonium transporters and members of the major intrinsic protein (MIP) family. The homo-tetrameric calcium-gated potassium channel MthK (PDB code 1LNQ, MCC 0.76) and Na^+^/K^+^ conducting channel (2AHY, MCC 0.52) fared particularly well with the majority of pore-lining residues correctly identified. False positive predictions were usually clustered close to the pore in in the Na^+^/K conducting channel while none were predicted in MthK (Figure 1). Of the monomeric channels, the 12 transmembrane helix glycerol-3-phosphate transporter was the best predicted with the majority of pore-lining residues correctly identified (1PW4, MCC 0.50), while the ammonia channel and transporter predictions were also impressive. In the case of the ammonia transporter AmtB (1XQF), two phenylalanine side chains constrict pore access from the periplasmic side, requiring conformational change for transient pore opening [28]. These highly conserved residues are correctly predicted by the SVM as are the majority of those that line the pore (MCC 0.49). Similar gating regions can be found in transmembrane helices 3 and 5 of the molybdate transporter ModB2C2 (2ONK, MCC 0.49) which contain clusters of highly conserved residues [29], many of which are also correctly predicted (Figure 1). In general, identifying pores within transporters was more difficult, possibly due to the narrower and more frequently obstructed translocation pathway, with the physicochemical properties of the pore-lining residues bearing less resemblance to those lining the unobstructed water-filled pores in channel proteins. In a number of the worst performing targets, low conservation in multiple sequence alignments was evident suggesting that evolutionary information was not effectively captured.

### Identifying pore-lining helices

By using the average raw SVM score to identify pore-lining helices, performance is markedly improved compared to identification of pore-lining residues with a MCC of 0.43 and precision/recall values of 0.74 and 0.35. This represents an overall accuracy of 72% although the test set does display a slight bias towards negative examples (151:182). The performance increase compared to a naïve approach requiring an optimal threshold of at least 7 polar residues to identify pore-lining helices (MCC 0.15, accuracy 55%) is also substantial. Seven targets had all their helices correctly identified, including two 6-transmembrane helix aquaporin proteins (1Z98, 3C02) although in the cases of such proteins with re-entrant helices, these regions were not included in the prediction. An attempt to include re-entrant helices resulted in lower overall performance, in part due to the difficulty in accurately predicting the location of these features. Cross-validated topology prediction was correct in 39 cases (75%), with a total of 5 over-predicted and 12 under-predicted in the remainder of cases. These results are lower than MEMSAT-SVM benchmark results most likely due to the difficulty in predicting pore-lining helices that contain a higher fraction of polar residues than average.

**Figure 2:**
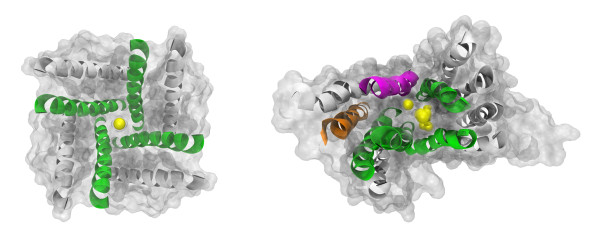
**Prediction of pore-lining helices from sequence, mapped onto native crystal structures.** Homotetrameric potassium channel Kir3.1 (left, 2QKS); both helices in each subunit are correctly classified. Monomeric 12 transmembrane helix multiple-drug resistance transporter NorM (right, 3MKT); 10 out of 12 helices are correctly classified–one false positive and one false negative are predicted. The pore identified by Pore-Walker is shown in yellow spheres, while helices correctly predicted as pore-lining are shown in green (true positives), incorrectly predicted as pore-lining are shown in orange (false positives), correctly predicted as non-pore-lining are shown in grey (true negatives) and incorrectly predicted as non-pore-lining are shown in magenta (false negatives). In both cases, all transmembrane helices were correctly predicted by MEMSAT-SVM.

In 30 cases (58%), at least 75% of helices were correctly identified. These included a number of large monomeric targets with complex topologies such as the 12 transmembrane helix multiple-drug resistance transporter NorM (3MKT) and ApcT transporter (3GIA) proteins where only two helices are misclassified in each case (Figure 2), while the 11 transmembrane helix ammonia transporters Amt-1 (2B2F) and Rh50 (3B9W) have one and two misclassified helices respectively. A range of multimeric targets including potassium, mechanosensitive and amiloride-sensitive cation channels had all helices correctly identified (Figure 2).

### Predicting pore stoichiometry

**Figure 3:**
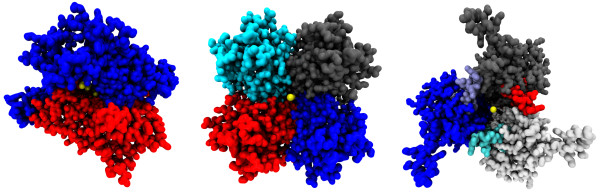
**Correctly predicted stoichiometries of three multimeric pore complexes.** Bottom-up view of the heterodimeric maltose transporter malFG (left, 2R6G chain F), homotetrameric inward rectifier potassium channel KirBac1.1 (centre, 1P7B) and heterohexameric formate dehydrogenase (right, 1KQG chain B). Stoichiometry relates to the pore-lining membrane region only–extramembranous chains or those that do not line the pore are excluded. Complexes are coloured by chain, with the Pore-Walker predicted location of the pore shown by yellow spheres.

Input features for the pore stoichiometry SVR were based on topology predicted by MEMSAT-SVM, and predicted pore-lining residues and helices as outlined above, all under full cross-validation, with output values rounded to the nearest integer. The model correctly predicted the pore stoichiometry for 32 targets (62%) with a Pearson correlation coefficient of 0.72 and average error of 0.75 subunits. Proteins forming monomeric pores in almost all cases contain three or more pore-lining helices and were generally easiest to identify, with only three false positive predictions. On this basis, proteins forming monomeric pores can be discriminated from those forming multimeric pores in 90% of cases. Of the 27 multimeric targets, 9 (33%) have their stoichiometry correctly determined including a number of tetrameric cation channels and various dimeric transporters including the multidrug ABC transporter SAV1866 (2HYD), molybdate transporter ModB2C2 (2ONK), maltose transport system permease protein malFG (2R6G) and the heterohexameric proton translocating formate dehydrogenase (1KQG chain B) (Figure 3). In a number of cases where stoichiometries of three or more subunits were observed in native structures, predictions differed by only a single subunit including the homotrimeric ATP-gated P2X4 ion channel (3H9V), predicted as tetramer, and the homotetrameric potassium channel NaK (2AHY) which was predicted as a pentamer. Under-prediction of pore-lining helices appears to have a significant effect on the prediction of pore stoichiometry, as 8 out of 11 cases are incorrect when no pore-lining helices are predicted (Table 2).

**Table 2:** Pore-lining residue, pore-lining helix, topology and pore stoichiometry results for all targets

**Target**	**Per-residue MCC**	**Pore-lining helices**		**Topology**		**Pore stoichiometry**	
		**Observed**	**Predicted**	**Correct**	**Over/Under**	**Observed**	**Predicted**
1E12_A	0.04	4	2	1	0	1	1
1FFT_A	0.28	3	3	0	3	1	1
1KQG_B	−0.42	1	0	0	0	6	6
1KQG_C	0.03	2	0	1	0	6	5
1L7V_B	0.63	2	2	0	1	2	1
1LDI_A	0.41	5	2	1	0	1	3
1LNQ_A	0.76	1	0	1	0	4	4
1P7B_A	0.52	1	1	1	0	4	4
1PW4_A	0.50	7	3	1	0	1	1
1R3J_C	0.54	1	0	1	0	4	6
1T9Y_A	0.31	6	2	0	−4	1	1
1XL6_A	0.51	1	1	1	0	4	4
1XME_A	0.27	3	3	1	0	1	1
1XQF_A	0.52	5	4	0	−1	1	1
1Z98_A	0.35	3	3	1	0	1	1
1ZLL_E	0.00	1	0	0	0	5	3
2AHY_A	0.52	1	0	1	0	4	5
2B2F_A	0.49	4	3	0	−2	1	1
2BG9_A	0.15	1	1	1	0	5	2
2BG9_E	0.37	1	1	1	0	5	2
2C3E_A	−0.10	6	3	0	−2	1	1
2C8L_A	0.37	4	3	1	0	1	1
2D57_A	0.28	4	3	1	0	1	1
2F2B_A	0.05	3	1	1	0	1	3
2GFP_A	0.24	6	2	1	0	1	1
2HYD_A	0.22	4	1	0	−1	2	2
2OAR_A	0.00	1	1	1	0	5	3
2OAU_A	0.11	1	0	1	0	7	4
2ONK_C	0.49	3	1	1	0	2	2
2QFI_A	0.26	2	1	1	0	2	3
2QKS_A	0.44	1	1	1	0	4	4
2QTS_A	0.50	1	1	1	0	3	5
2R6G_F	0.33	2	1	1	0	2	2
2R6G_G	0.07	3	1	1	0	2	1
2RDD_B	−0.61	1	0	0	0	3	5
2W2E_A	0.38	4	3	1	0	1	1
2ZW3_A	−0.36	1	0	1	0	6	3
3B8C_A	0.45	5	3	1	0	1	1
3B9W_A	0.46	5	4	1	0	1	1
3BEH_A	0.22	1	0	0	−1	4	2
3C02_A	0.37	4	4	1	0	1	1
3DDL_A	0.11	4	2	1	0	1	1
3DHW_A	0.20	2	1	1	0	2	2
3EAM_A	0.30	1	0	0	−1	5	3
3GIA_A	0.32	4	3	1	0	1	1
3H9V_A	0.13	2	1	1	0	3	4
3HD6_A	0.50	4	4	1	0	1	1
3K3F_A	0.44	4	2	1	0	1	1
3KG2_A	0.37	1	1	0	1	4	1
3MKT_A	0.32	5	4	1	0	1	1
3O0R_B	0.11	5	0	1	0	1	1
3P5N_A	0.10	4	3	1	0	1	1

A correct topology (requiring the correct number and placement of all transmembrane helices but not N-terminus) is indicated by 1, and otherwise 0. Over/Under corresponds to the number of over-predicted or under-predicted transmembrane helices.

## Conclusions

We have developed a novel method capable of automatically detecting pores in alpha-helical transmembrane proteins using sequence information alone. Validation of this approach by testing on a data set of 52 protein chains has demonstrated that pore-lining helices can be detected with an accuracy of 72% and a precision as high as 0.74. While these results are encouraging, the low number of chains in the training set is likely to have limited SVM performance since small data sets reduce tolerance to errors and the ability of SVMs to develop large generalisation bounds. Additionally, the use of crystal structures to determine pore-lining residues neglects the inherent dynamic nature of membrane proteins which exhibit significant conformational flexibility. Transporters, in particular, undergo conformational changes within the membrane region to transport the substrate across the bilayer, and this may account for the relative difficulty in identifying pore-lining residues compared to channel proteins, where the gating mechanism usually lies at the membrane/solvent interface, while causing labelling errors when analysing the structure using Pore-Walker. A more suitable approach might be to extract structures at regular intervals during molecular dynamic simulations before analysis with Pore-Walker, and perhaps excluding transporters unless both open and closed conformations are available. SVR prediction of pore stoichiometry suggests that discrimination between monomeric and multimeric pores is possible with a high degree of accuracy. However, predicting the exact stoichiometry of multimeric pores remains a challenge with only a third of cases successful. Again, the relatively limited training set is likely to be a hindrance while a more diverse range of input features, perhaps including global estimates of solvent accessibility and lipid exposure, may improve performance.

Despite these challenges, the successful identification of pores and channels in uncharacterised membrane protein sequences, as well as estimates of their stoichiometries, may be of substantial biochemical and pharmacological significance. A large number of channelopathies, diseases caused by disturbed function of ion channel subunits, have already been identified and tools to characterise pores in disease-related proteins may provide valuable insight into routes of therapeutic intervention. From a structural modelling perspective, both identification of pore-forming regions and complex stoichiometry may provide important insight into quaternary structure geometry and assist *de novo* methods in modelling such regions so that they are solvent accessible rather than lipid embedded. Furthermore, site-directed mutagenesis of predicted pore-lining residues may allow modification of substrate specificity, providing valuable insight into protein design.

### Availability

Pore-identification has been integrated into our transmembrane topology predictor MEMSAT-SVM and is available as source code from the URL below and is free for non-commercial use. All data sets are also available, and cross-validation SVM model files are available on request. The software has been tested on a Linux operating system. In order to compile and run, the gcc compiler, Perl interpreter and NCBI tools are required.

http://bioinf.cs.ucl.ac.uk/downloads/memsat-svm/.

## Competing interests

The authors declare that they have no competing interests.

## Authors’ contributions

Both authors have contributed equally to this work. Source code was developed by TN. DTJ provided direction for computational aspects of the algorithm and biological/biophysical insight into aspects of membrane protein structure. Manuscript was prepared by TN and has been edited and approved by DTJ. Both authors read and approved the final manuscript.
